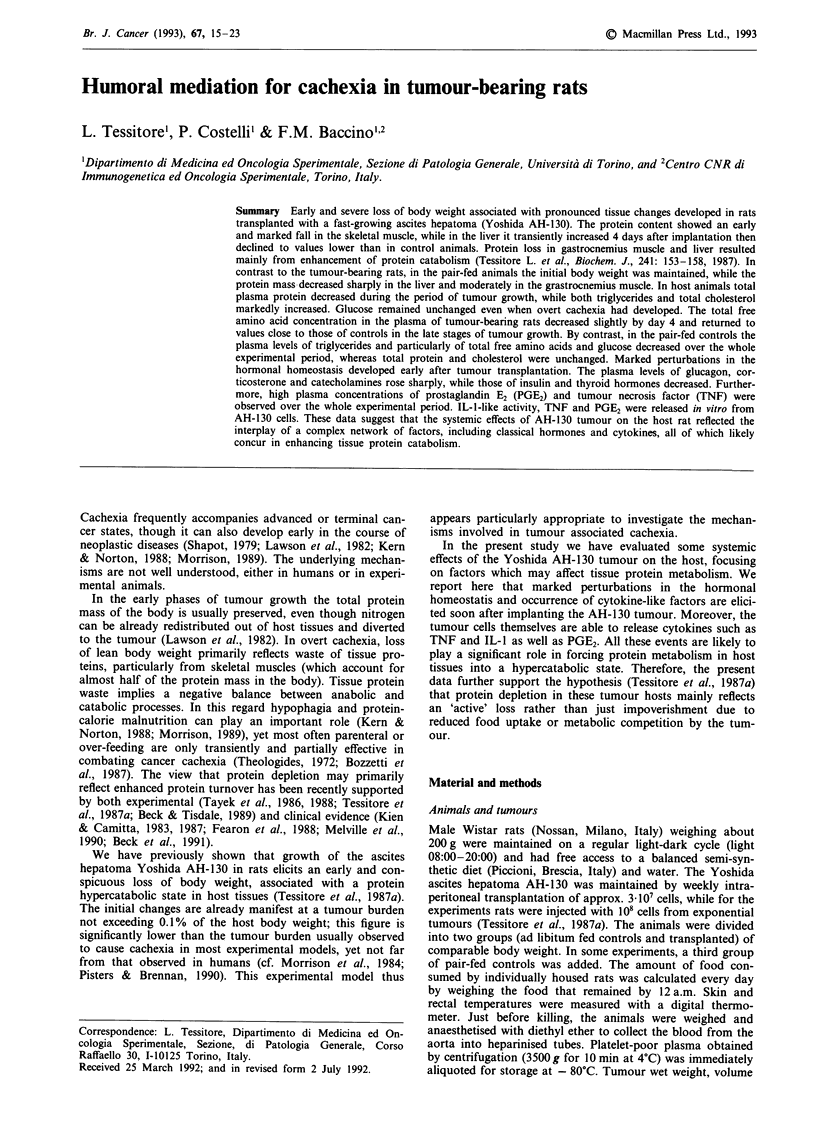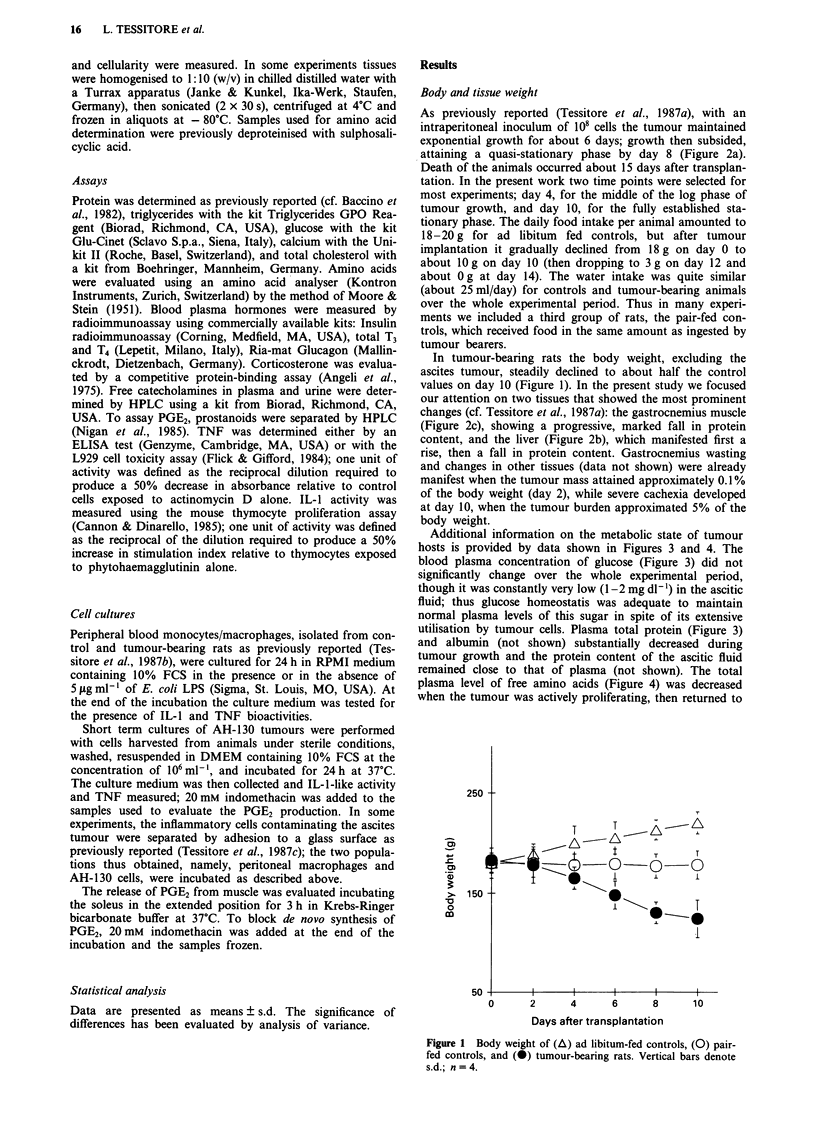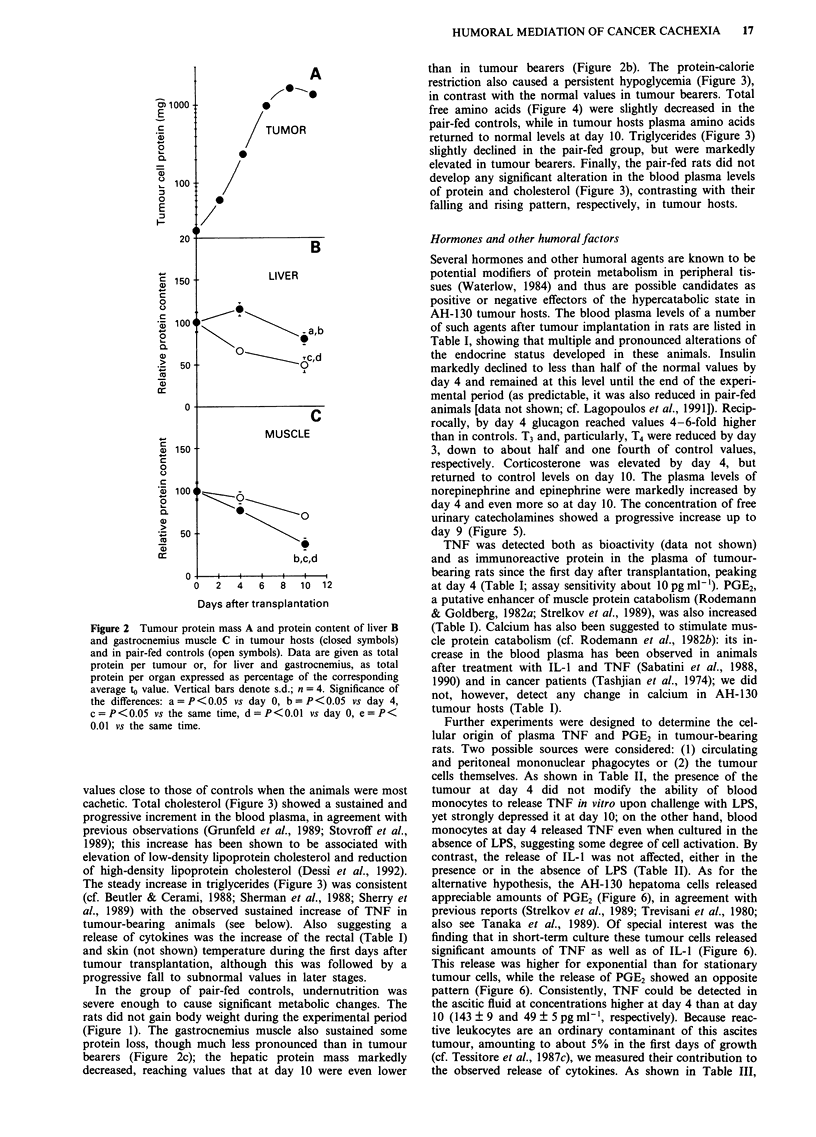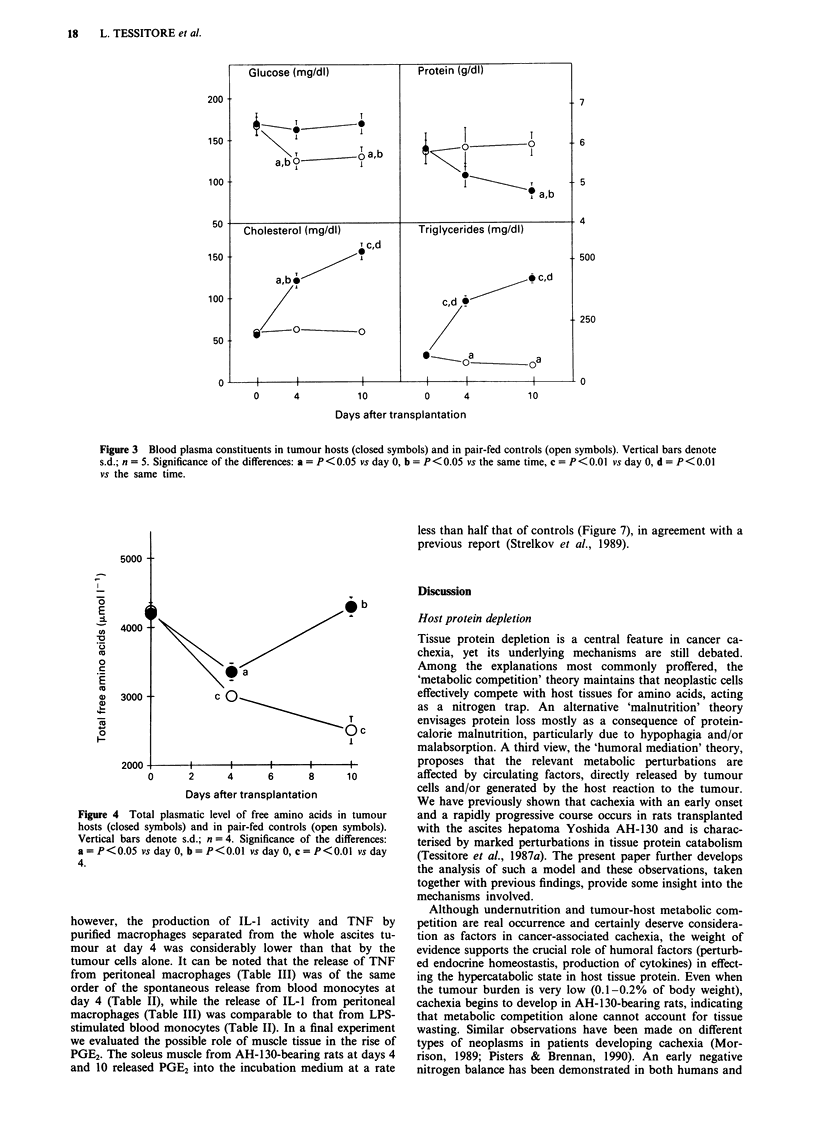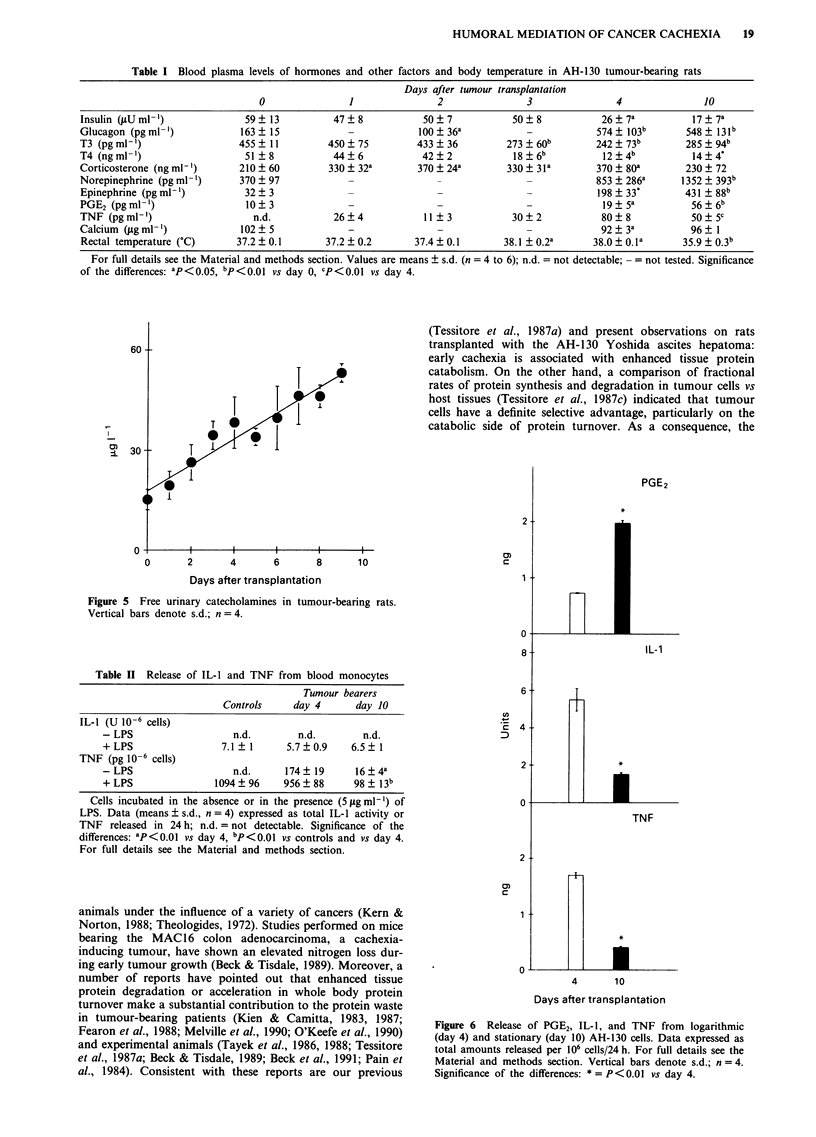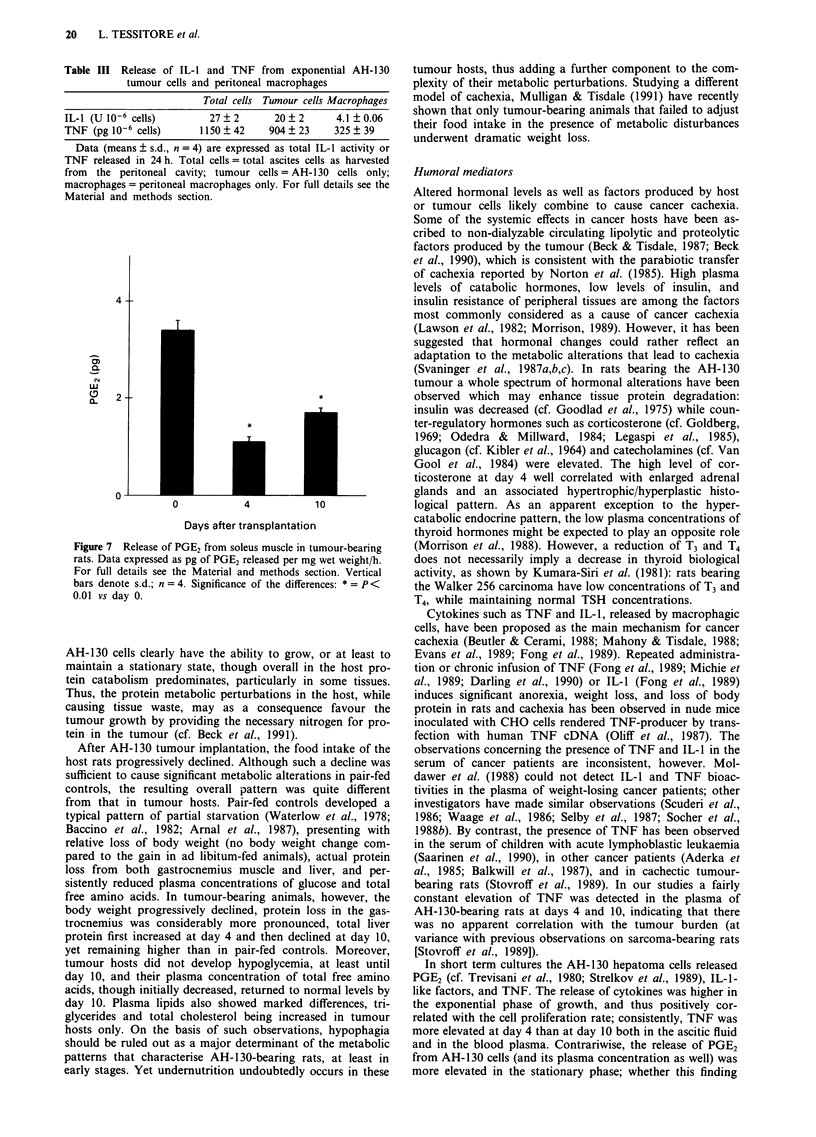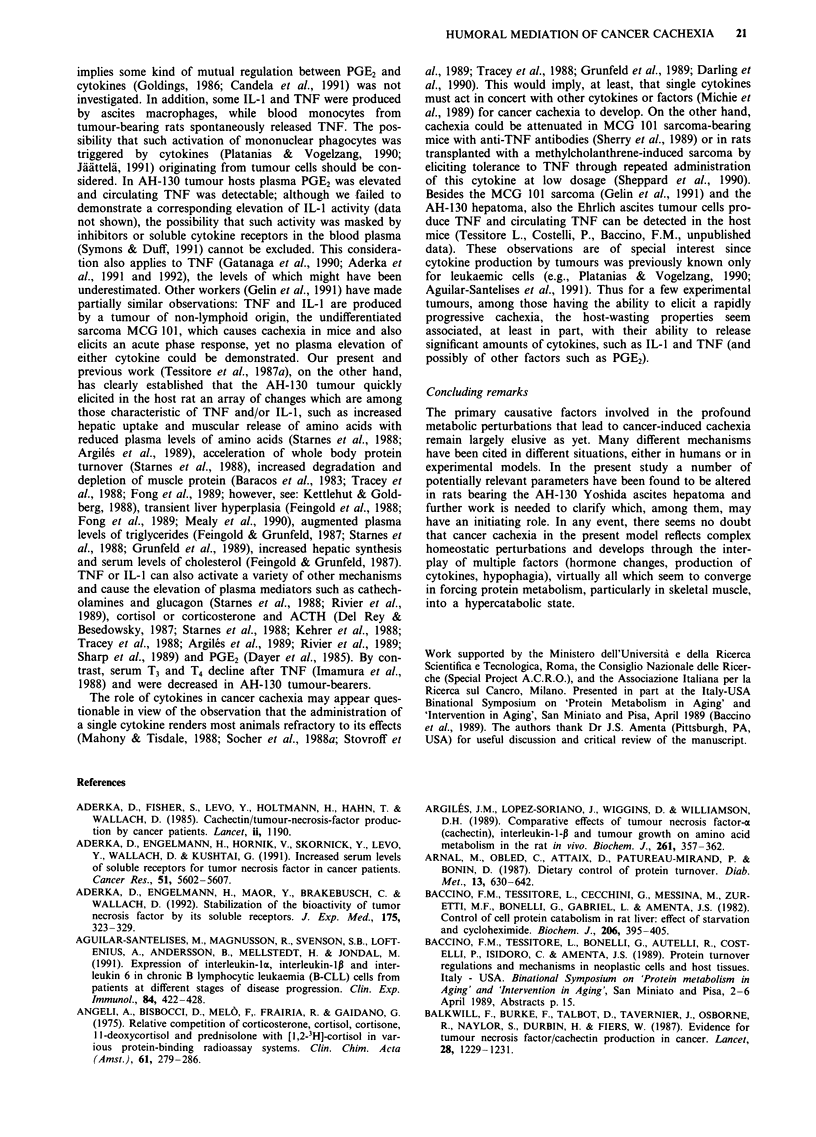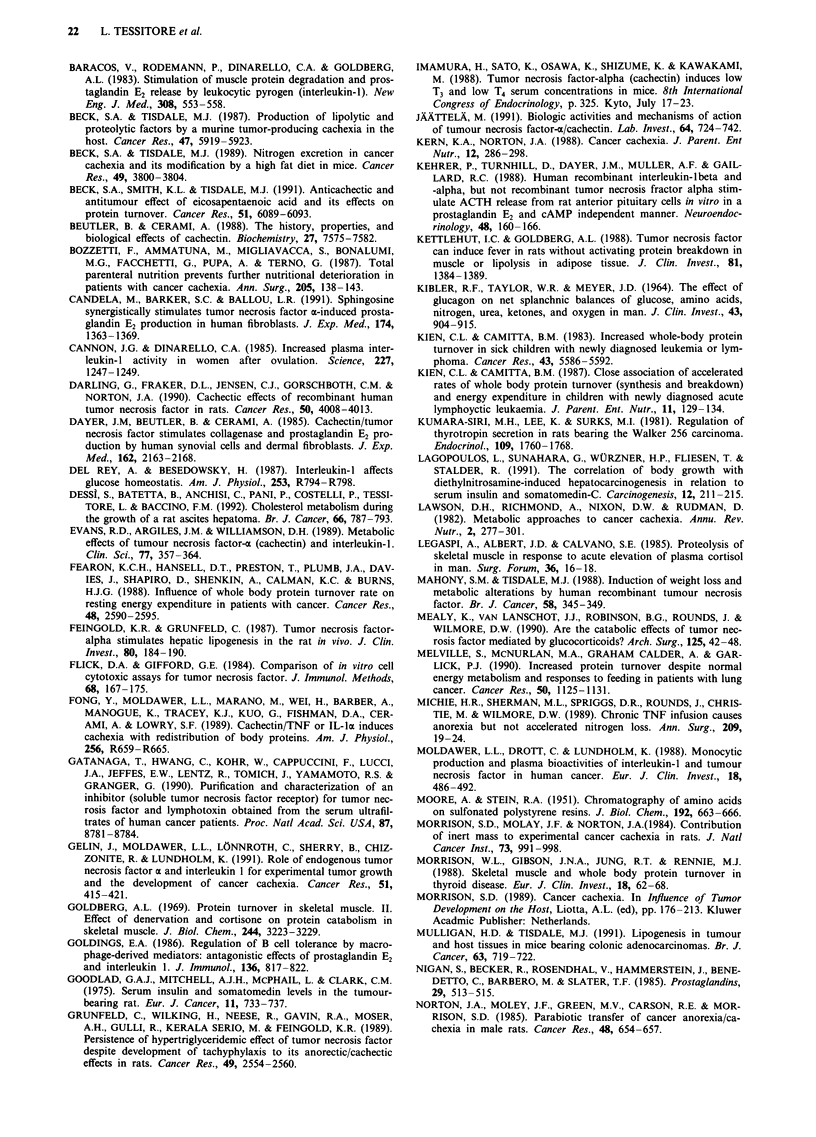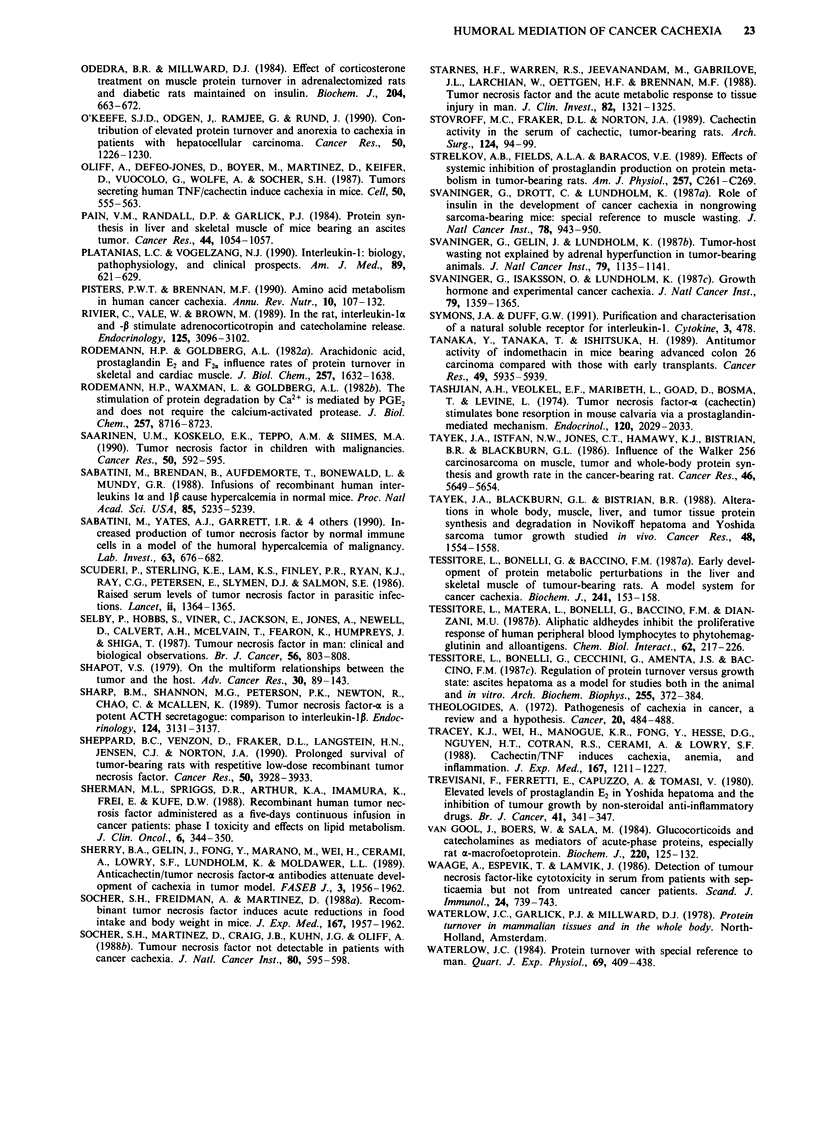# Humoral mediation for cachexia in tumour-bearing rats.

**DOI:** 10.1038/bjc.1993.4

**Published:** 1993-01

**Authors:** L. Tessitore, P. Costelli, F. M. Baccino

**Affiliations:** Dipartimento di Medicina ed Oncologia Sperimentale, Università di Torino, Italy.

## Abstract

Early and severe loss of body weight associated with pronounced tissue changes developed in rats transplanted with a fast-growing ascites hepatoma (Yoshida AH-130). The protein content showed an early and marked fall in the skeletal muscle, while in the liver it transiently increased 4 days after implantation then declined to values lower than in control animals. Protein loss in gastrocnemius muscle and liver resulted mainly from enhancement of protein catabolism (Tessitore L. et al., Biochem. J., 241: 153-158, 1987). In contrast to the tumour-bearing rats, in the pair-fed animals the initial body weight was maintained, while the protein mass decreased sharply in the liver and moderately in the gastrocnemius muscle. In host animals total plasma protein decreased during the period of tumour growth, while both triglycerides and total cholesterol markedly increased. Glucose remained unchanged even when overt cachexia had developed. The total free amino acid concentration in the plasma of tumour-bearing rats decreased slightly by day 4 and returned to values close to those of controls in the late stages of tumour growth. By contrast, in the pair-fed controls the plasma levels of triglycerides and particularly of total free amino acids and glucose decreased over the whole experimental period, whereas total protein and cholesterol were unchanged. Marked perturbations in the hormonal homeostasis developed early after tumour transplantation. The plasma levels of glucagon, corticosterone and catecholamines rose sharply, while those of insulin and thyroid hormones decreased. Furthermore, high plasma concentrations of prostaglandin E2 (PGE2) and tumour necrosis factor (TNF) were observed over the whole experimental period. IL-1-like activity, TNF and PGE2 were released in vitro from AH-130 cells. These data suggest that the systemic effects of AH-130 tumour on the host rat reflected the interplay of a complex network of factors, including classical hormones and cytokines, all of which likely concur in enhancing tissue protein catabolism.


Br. J. Cancer (1993), 67, 15–23 © Macmillan Press Ltd., 1993

### Humoral mediation for cachexia in tumour-bearing rats

L. Tessitore[1], P. Costelli[1] & F.M. Baccino[1,2]

[1]*Dipartimento di Medicina ed Oncologia Sperimentale, Sezione di Patologia Generale, Università di Torino, and* [2]*Centro CNR di Immunogenetica ed Oncologia Sperimentale, Torino, Italy.*

**Summary** Early and severe loss of body weight associated with pronounced tissue changes developed in rats transplanted with a fast-growing ascites hepatoma (Yoshida AH-130). The protein content showed an early and marked fall in the skeletal muscle, while in the liver it transiently increased 4 days after implantation then declined to values lower than in control animals. Protein loss in gastrocnemius muscle and liver resulted mainly from enhancement of protein catabolism (Tessitore L. *et al.*, *Biochem. J.*, 241: 153–158, 1987). In contrast to the tumour-bearing rats, in the pair-fed animals the initial body weight was maintained, while the protein mass decreased sharply in the liver and moderately in the grastrocnemius muscle. In host animals total plasma protein decreased during the period of tumour growth, while both triglycerides and total cholesterol markedly increased. Glucose remained unchanged even when overt cachexia had developed. The total free amino acid concentration in the plasma of tumour-bearing rats decreased slightly by day 4 and returned to values close to those of controls in the late stages of tumour growth. By contrast, in the pair-fed controls the plasma levels of triglycerides and particularly of total free amino acids and glucose decreased over the whole experimental period, whereas total protein and cholesterol were unchanged. Marked perturbations in the hormonal homeostasis developed early after tumour transplantation. The plasma levels of glucagon, corticosterone and catecholamines rose sharply, while those of insulin and thyroid hormones decreased. Furthermore, high plasma concentrations of prostaglandin $E_2$ ($PGE_2$) and tumour necrosis factor (TNF) were observed over the whole experimental period. IL-1-like activity, TNF and $PGE_2$ were released *in vitro* from AH-130 cells. These data suggest that the systemic effects of AH-130 tumour on the host rat reflected the interplay of a complex network of factors, including classical hormones and cytokines, all of which likely concur in enhancing tissue protein catabolism.

Cachexia frequently accompanies advanced or terminal cancer states, though it can also develop early in the course of neoplastic diseases (Shapot, 1979; Lawson *et al.*, 1982; Kern & Norton, 1988; Morrison, 1989). The underlying mechanisms are not well understood, either in humans or in experimental animals.

In the early phases of tumour growth the total protein mass of the body is usually preserved, even though nitrogen can be already redistributed out of host tissues and diverted to the tumour (Lawson *et al.*, 1982). In overt cachexia, loss of lean body weight primarily reflects waste of tissue proteins, particularly from skeletal muscles (which account for almost half of the protein mass in the body). Tissue protein waste implies a negative balance between anabolic and catabolic processes. In this regard hypophagia and protein-calorie malnutrition can play an important role (Kern & Norton, 1988; Morrison, 1989), yet most often parenteral or over-feeding are only transiently and partially effective in combating cancer cachexia (Theologides, 1972; Bozzetti *et al.*, 1987). The view that protein depletion may primarily reflect enhanced protein turnover has been recently supported by both experimental (Tayek *et al.*, 1986, 1988; Tessitore *et al.*, 1987*a*; Beck & Tisdale, 1989) and clinical evidence (Kien & Camitta, 1983, 1987; Fearon *et al.*, 1988; Melville *et al.*, 1990; Beck *et al.*, 1991).

We have previously shown that growth of the ascites hepatoma Yoshida AH-130 in rats elicits an early and conspicuous loss of body weight, associated with a protein hypercatabolic state in host tissues (Tessitore *et al.*, 1987*a*). The initial changes are already manifest at a tumour burden not exceeding 0.1% of the host body weight; this figure is significantly lower than the tumour burden usually observed to cause cachexia in most experimental models, yet not far from that observed in humans (cf. Morrison *et al.*, 1984; Pisters & Brennan, 1990). This experimental model thus appears particularly appropriate to investigate the mechanisms involved in tumour associated cachexia.

In the present study we have evaluated some systemic effects of the Yoshida AH-130 tumour on the host, focusing on factors which may affect tissue protein metabolism. We report here that marked perturbations in the hormonal homeostatis and occurrence of cytokine-like factors are elicited soon after implanting the AH-130 tumour. Moreover, the tumour cells themselves are able to release cytokines such as TNF and IL-1 as well as $PGE_2$. All these events are likely to play a significant role in forcing protein metabolism in host tissues into a hypercatabolic state. Therefore, the present data further support the hypothesis (Tessitore *et al.*, 1987*a*) that protein depletion in these tumour hosts mainly reflects an 'active' loss rather than just impoverishment due to reduced food uptake or metabolic competition by the tumour.

### Material and methods

### *Animals and tumours*

Male Wistar rats (Nossan, Milano, Italy) weighing about 200 g were maintained on a regular light-dark cycle (light 08:00–20:00) and had free access to a balanced semi-synthetic diet (Piccioni, Brescia, Italy) and water. The Yoshida ascites hepatoma AH-130 was maintained by weekly intraperitoneal transplantation of approx. $3 \cdot 10^7$ cells, while for the experiments rats were injected with $10^8$ cells from exponential tumours (Tessitore *et al.*, 1987*a*). The animals were divided into two groups (ad libitum fed controls and transplanted) of comparable body weight. In some experiments, a third group of pair-fed controls was added. The amount of food consumed by individually housed rats was calculated every day by weighing the food that remained by 12 a.m. Skin and rectal temperatures were measured with a digital thermometer. Just before killing, the animals were weighed and anaesthetised with diethyl ether to collect the blood from the aorta into heparinised tubes. Platelet-poor plasma obtained by centrifugation (3500 *g* for 10 min at 4°C) was immediately aliquoted for storage at − 80°C. Tumour wet weight, volume

Correspondence: L. Tessitore, Dipartimento di Medicina ed Oncologia Sperimentale, Sezione, di Patologia Generale, Corso Raffaello 30, I-10125 Torino, Italy.
Received 25 March 1992; and in revised form 2 July 1992.

and cellularity were measured. In some experiments tissues were homogenised to 1:10 (w/v) in chilled distilled water with a Turrax apparatus (Janke & Kunkel, Ika-Werk, Staufen, Germany), then sonicated (2 × 30 s), centrifuged at 4°C and frozen in aliquots at − 80°C. Samples used for amino acid determination were previously deproteinised with sulphosalicyclic acid.

*Assays*

Protein was determined as previously reported (cf. Baccino *et al.*, 1982), triglycerides with the kit Triglycerides GPO Reagent (Biorad, Richmond, CA, USA), glucose with the kit Glu-Cinet (Sclavo S.p.a., Siena, Italy), calcium with the Unikit II (Roche, Basel, Switzerland), and total cholesterol with a kit from Boehringer, Mannheim, Germany. Amino acids were evaluated using an amino acid analyser (Kontron Instruments, Zurich, Switzerland) by the method of Moore & Stein (1951). Blood plasma hormones were measured by radioimmunoassay using commercially available kits: Insulin radioimmunoassay (Corning, Medfield, MA, USA), total $T_3$ and $T_4$ (Lepetit, Milano, Italy), Ria-mat Glucagon (Mallinckrodt, Dietzenbach, Germany). Corticosterone was evaluated by a competitive protein-binding assay (Angeli *et al.*, 1975). Free catecholamines in plasma and urine were determined by HPLC using a kit from Biorad, Richmond, CA, USA. To assay $PGE_2$, prostanoids were separated by HPLC (Nigan *et al.*, 1985). TNF was determined either by an ELISA test (Genzyme, Cambridge, MA, USA) or with the L929 cell toxicity assay (Flick & Gifford, 1984); one unit of activity was defined as the reciprocal dilution required to produce a 50% decrease in absorbance relative to control cells exposed to actinomycin D alone. IL-1 activity was measured using the mouse thymocyte proliferation assay (Cannon & Dinarello, 1985); one unit of activity was defined as the reciprocal of the dilution required to produce a 50% increase in stimulation index relative to thymocytes exposed to phytohaemagglutinin alone.

*Cell cultures*

Peripheral blood monocytes/macrophages, isolated from control and tumour-bearing rats as previously reported (Tessitore *et al.*, 1987*b*), were cultured for 24 h in RPMI medium containing 10% FCS in the presence or in the absence of 5 $\mu g\ ml^{-1}$ of *E. coli* LPS (Sigma, St. Louis, MO, USA). At the end of the incubation the culture medium was tested for the presence of IL-1 and TNF bioactivities.

Short term cultures of AH-130 tumours were performed with cells harvested from animals under sterile conditions, washed, resuspended in DMEM containing 10% FCS at the concentration of $10^6\ ml^{-1}$, and incubated for 24 h at 37°C. The culture medium was then collected and IL-1-like activity and TNF measured; 20 mM indomethacin was added to the samples used to evaluate the $PGE_2$ production. In some experiments, the inflammatory cells contaminating the ascites tumour were separated by adhesion to a glass surface as previously reported (Tessitore *et al.*, 1987*c*); the two populations thus obtained, namely, peritoneal macrophages and AH-130 cells, were incubated as described above.

The release of $PGE_2$ from muscle was evaluated incubating the soleus in the extended position for 3 h in Krebs-Ringer bicarbonate buffer at 37°C. To block *de novo* synthesis of $PGE_2$, 20 mM indomethacin was added at the end of the incubation and the samples frozen.

*Statistical analysis*

Data are presented as means ± s.d. The significance of differences has been evaluated by analysis of variance.

**Results**

*Body and tissue weight*

As previously reported (Tessitore *et al.*, 1987*a*), with an intraperitoneal inoculum of $10^8$ cells the tumour maintained exponential growth for about 6 days; growth then subsided, attaining a quasi-stationary phase by day 8 (Figure 2a). Death of the animals occurred about 15 days after transplantation. In the present work two time points were selected for most experiments; day 4, for the middle of the log phase of tumour growth, and day 10, for the fully established stationary phase. The daily food intake per animal amounted to 18–20 g for ad libitum fed controls, but after tumour implantation it gradually declined from 18 g on day 0 to about 10 g on day 10 (then dropping to 3 g on day 12 and about 0 g at day 14). The water intake was quite similar (about 25 ml/day) for controls and tumour-bearing animals over the whole experimental period. Thus in many experiments we included a third group of rats, the pair-fed controls, which received food in the same amount as ingested by tumour bearers.

In tumour-bearing rats the body weight, excluding the ascites tumour, steadily declined to about half the control values on day 10 (Figure 1). In the present study we focused our attention on two tissues that showed the most prominent changes (cf. Tessitore *et al.*, 1987*a*): the gastrocnemius muscle (Figure 2c), showing a progressive, marked fall in protein content, and the liver (Figure 2b), which manifested first a rise, then a fall in protein content. Gastrocnemius wasting and changes in other tissues (data not shown) were already manifest when the tumour mass attained approximately 0.1% of the body weight (day 2), while severe cachexia developed at day 10, when the tumour burden approximated 5% of the body weight.

Additional information on the metabolic state of tumour hosts is provided by data shown in Figures 3 and 4. The blood plasma concentration of glucose (Figure 3) did not significantly change over the whole experimental period, though it was constantly very low (1–2 $mg\ dl^{-1}$) in the ascitic fluid; thus glucose homeostatis was adequate to maintain normal plasma levels of this sugar in spite of its extensive utilisation by tumour cells. Plasma total protein (Figure 3) and albumin (not shown) substantially decreased during tumour growth and the protein content of the ascitic fluid remained close to that of plasma (not shown). The total plasma level of free amino acids (Figure 4) was decreased when the tumour was actively proliferating, then returned to

**Figure 1** Body weight of (Δ) ad libitum-fed controls, (O) pair-fed controls, and (●) tumour-bearing rats. Vertical bars denote s.d.; $n = 4$.

**Figure 2** Tumour protein mass **A** and protein content of liver **B** and gastrocnemius muscle **C** in tumour hosts (closed symbols) and in pair-fed controls (open symbols). Data are given as total protein per tumour or, for liver and gastrocnemius, as total protein per organ expressed as percentage of the corresponding average $t_0$ value. Vertical bars denote s.d.; $n = 4$. Significance of the differences: a = $P < 0.05$ *vs* day 0, b = $P < 0.05$ *vs* day 4, c = $P < 0.05$ *vs* the same time, d = $P < 0.01$ *vs* day 0, e = $P <$ 0.01 *vs* the same time.

values close to those of controls when the animals were most cachetic. Total cholesterol (Figure 3) showed a sustained and progressive increment in the blood plasma, in agreement with previous observations (Grunfeld *et al.*, 1989; Stovroff *et al.*, 1989); this increase has been shown to be associated with elevation of low-density lipoprotein cholesterol and reduction of high-density lipoprotein cholesterol (Dessi *et al.*, 1992). The steady increase in triglycerides (Figure 3) was consistent (cf. Beutler & Cerami, 1988; Sherman *et al.*, 1988; Sherry *et al.*, 1989) with the observed sustained increase of TNF in tumour-bearing animals (see below). Also suggesting a release of cytokines was the increase of the rectal (Table I) and skin (not shown) temperature during the first days after tumour transplantation, although this was followed by a progressive fall to subnormal values in later stages.

In the group of pair-fed controls, undernutrition was severe enough to cause significant metabolic changes. The rats did not gain body weight during the experimental period (Figure 1). The gastrocnemius muscle also sustained some protein loss, though much less pronounced than in tumour bearers (Figure 2c); the hepatic protein mass markedly decreased, reaching values that at day 10 were even lower than in tumour bearers (Figure 2b). The protein-calorie restriction also caused a persistent hypoglycemia (Figure 3), in contrast with the normal values in tumour bearers. Total free amino acids (Figure 4) were slightly decreased in the pair-fed controls, while in tumour hosts plasma amino acids returned to normal levels at day 10. Triglycerides (Figure 3) slightly declined in the pair-fed group, but were markedly elevated in tumour bearers. Finally, the pair-fed rats did not develop any significant alteration in the blood plasma levels of protein and cholesterol (Figure 3), contrasting with their falling and rising pattern, respectively, in tumour hosts.

*Hormones and other humoral factors*

Several hormones and other humoral agents are known to be potential modifiers of protein metabolism in peripheral tissues (Waterlow, 1984) and thus are possible candidates as positive or negative effectors of the hypercatabolic state in AH-130 tumour hosts. The blood plasma levels of a number of such agents after tumour implantation in rats are listed in Table I, showing that multiple and pronounced alterations of the endocrine status developed in these animals. Insulin markedly declined to less than half of the normal values by day 4 and remained at this level until the end of the experimental period (as predictable, it was also reduced in pair-fed animals [data not shown; cf. Lagopoulos *et al.*, 1991]). Reciprocally, by day 4 glucagon reached values 4–6-fold higher than in controls. $T_3$ and, particularly, $T_4$ were reduced by day 3, down to about half and one fourth of control values, respectively. Corticosterone was elevated by day 4, but returned to control levels on day 10. The plasma levels of norepinephrine and epinephrine were markedly increased by day 4 and even more so at day 10. The concentration of free urinary catecholamines showed a progressive increase up to day 9 (Figure 5).

TNF was detected both as bioactivity (data not shown) and as immunoreactive protein in the plasma of tumour-bearing rats since the first day after transplantation, peaking at day 4 (Table I; assay sensitivity about 10 pg $ml^{-1}$). $PGE_2$, a putative enhancer of muscle protein catabolism (Rodemann & Goldberg, 1982*a*; Strelkov *et al.*, 1989), was also increased (Table I). Calcium has also been suggested to stimulate muscle protein catabolism (cf. Rodemann *et al.*, 1982*b*): its increase in the blood plasma has been observed in animals after treatment with IL-1 and TNF (Sabatini *et al.*, 1988, 1990) and in cancer patients (Tashjian *et al.*, 1974); we did not, however, detect any change in calcium in AH-130 tumour hosts (Table I).

Further experiments were designed to determine the cellular origin of plasma TNF and $PGE_2$ in tumour-bearing rats. Two possible sources were considered: (1) circulating and peritoneal mononuclear phagocytes or (2) the tumour cells themselves. As shown in Table II, the presence of the tumour at day 4 did not modify the ability of blood monocytes to release TNF *in vitro* upon challenge with LPS, yet strongly depressed it at day 10; on the other hand, blood monocytes at day 4 released TNF even when cultured in the absence of LPS, suggesting some degree of cell activation. By contrast, the release of IL-1 was not affected, either in the presence or in the absence of LPS (Table II). As for the alternative hypothesis, the AH-130 hepatoma cells released appreciable amounts of $PGE_2$ (Figure 6), in agreement with previous reports (Strelkov *et al.*, 1989; Trevisani *et al.*, 1980; also see Tanaka *et al.*, 1989). Of special interest was the finding that in short-term culture these tumour cells released significant amounts of TNF as well as of IL-1 (Figure 6). This release was higher for exponential than for stationary tumour cells, while the release of $PGE_2$ showed an opposite pattern (Figure 6). Consistently, TNF could be detected in the ascitic fluid at concentrations higher at day 4 than at day 10 (143 ± 9 and 49 ± 5 pg $ml^{-1}$, respectively). Because reactive leukocytes are an ordinary contaminant of this ascites tumour, amounting to about 5% in the first days of growth (cf. Tessitore *et al.*, 1987*c*), we measured their contribution to the observed release of cytokines. As shown in Table III,

**Figure 3** Blood plasma constituents in tumour hosts (closed symbols) and in pair-fed controls (open symbols). Vertical bars denote s.d.; $n = 5$. Significance of the differences: **a** = $P<0.05$ *vs* day 0, **b** = $P<0.05$ *vs* the same time, **c** = $P<0.01$ *vs* day 0, **d** = $P<0.01$ *vs* the same time.

**Figure 4** Total plasmatic level of free amino acids in tumour hosts (closed symbols) and in pair-fed controls (open symbols). Vertical bars denote s.d.; $n = 4$. Significance of the differences: **a** = $P<0.05$ *vs* day 0, **b** = $P<0.01$ *vs* day 0, **c** = $P<0.01$ *vs* day 4.

however, the production of IL-1 activity and TNF by purified macrophages separated from the whole ascites tumour at day 4 was considerably lower than that by the tumour cells alone. It can be noted that the release of TNF from peritoneal macrophages (Table III) was of the same order of the spontaneous release from blood monocytes at day 4 (Table II), while the release of IL-1 from peritoneal macrophages (Table III) was comparable to that from LPS-stimulated blood monocytes (Table II). In a final experiment we evaluated the possible role of muscle tissue in the rise of $PGE_2$. The soleus muscle from AH-130-bearing rats at days 4 and 10 released $PGE_2$ into the incubation medium at a rate less than half that of controls (Figure 7), in agreement with a previous report (Strelkov *et al.*, 1989).

### Discussion

### *Host protein depletion*

Tissue protein depletion is a central feature in cancer cachexia, yet its underlying mechanisms are still debated. Among the explanations most commonly proffered, the 'metabolic competition' theory maintains that neoplastic cells effectively compete with host tissues for amino acids, acting as a nitrogen trap. An alternative 'malnutrition' theory envisages protein loss mostly as a consequence of protein-calorie malnutrition, particularly due to hypophagia and/or malabsorption. A third view, the 'humoral mediation' theory, proposes that the relevant metabolic perturbations are affected by circulating factors, directly released by tumour cells and/or generated by the host reaction to the tumour. We have previously shown that cachexia with an early onset and a rapidly progressive course occurs in rats transplanted with the ascites hepatoma Yoshida AH-130 and is characterised by marked perturbations in tissue protein catabolism (Tessitore *et al.*, 1987*a*). The present paper further develops the analysis of such a model and these observations, taken together with previous findings, provide some insight into the mechanisms involved.

Although undernutrition and tumour-host metabolic competition are real occurrence and certainly deserve consideration as factors in cancer-associated cachexia, the weight of evidence supports the crucial role of humoral factors (perturbed endocrine homeostastis, production of cytokines) in effecting the hypercatabolic state in host tissue protein. Even when the tumour burden is very low (0.1–0.2% of body weight), cachexia begins to develop in AH-130-bearing rats, indicating that metabolic competition alone cannot account for tissue wasting. Similar observations have been made on different types of neoplasms in patients developing cachexia (Morrison, 1989; Pisters & Brennan, 1990). An early negative nitrogen balance has been demonstrated in both humans and

**Table I** Blood plasma levels of hormones and other factors and body temperature in AH-130 tumour-bearing rats

| | *Days after tumour transplantation* | | | | | |
|---|---|---|---|---|---|---|
| | *0* | *1* | *2* | *3* | *4* | *10* |
| Insulin (μU ml$^{-1}$) | 59 ± 13 | 47 ± 8 | 50 ± 7 | 50 ± 8 | 26 ± 7[a] | 17 ± 7[a] |
| Glucagon (pg ml$^{-1}$) | 163 ± 15 | – | 100 ± 36[a] | – | 574 ± 103[b] | 548 ± 131[b] |
| T3 (pg ml$^{-1}$) | 455 ± 11 | 450 ± 75 | 433 ± 36 | 273 ± 60[b] | 242 ± 73[b] | 285 ± 94[b] |
| T4 (ng ml$^{-1}$) | 51 ± 8 | 44 ± 6 | 42 ± 2 | 18 ± 6[b] | 12 ± 4[b] | 14 ± 4[*] |
| Corticosterone (ng ml$^{-1}$) | 210 ± 60 | 330 ± 32[a] | 370 ± 24[a] | 330 ± 31[a] | 370 ± 80[a] | 230 ± 72 |
| Norepinephrine (pg ml$^{-1}$) | 370 ± 97 | – | – | – | 853 ± 286[a] | 1352 ± 393[b] |
| Epinephrine (pg ml$^{-1}$) | 32 ± 3 | – | – | – | 198 ± 33[*] | 431 ± 88[b] |
| $PGE_2$ (pg ml$^{-1}$) | 10 ± 3 | – | – | – | 19 ± 5[a] | 56 ± 6[b] |
| TNF (pg ml$^{-1}$) | n.d. | 26 ± 4 | 11 ± 3 | 30 ± 2 | 80 ± 8 | 50 ± 5[c] |
| Calcium (μg ml$^{-1}$) | 102 ± 5 | – | – | – | 92 ± 3[a] | 96 ± 1 |
| Rectal temperature (°C) | 37.2 ± 0.1 | 37.2 ± 0.2 | 37.4 ± 0.1 | 38.1 ± 0.2[a] | 38.0 ± 0.1[a] | 35.9 ± 0.3[b] |

For full details see the Material and methods section. Values are means ± s.d. ($n = 4$ to 6); n.d. = not detectable; – = not tested. Significance of the differences: [a]$P<0.05$, [b]$P<0.01$ *vs* day 0, [c]$P<0.01$ *vs* day 4.

**Figure 5** Free urinary catecholamines in tumour-bearing rats. Vertical bars denote s.d.; $n = 4$.

**Table II** Release of IL-1 and TNF from blood monocytes

| | | *Tumour bearers* | |
|---|---|---|---|
| | *Controls* | *day 4* | *day 10* |
| IL-1 (U $10^{-6}$ cells) | | | |
| – LPS | n.d. | n.d. | n.d. |
| + LPS | 7.1 ± 1 | 5.7 ± 0.9 | 6.5 ± 1 |
| TNF (pg $10^{-6}$ cells) | | | |
| – LPS | n.d. | 174 ± 19 | 16 ± 4[a] |
| + LPS | 1094 ± 96 | 956 ± 88 | 98 ± 13[b] |

Cells incubated in the absence or in the presence (5 μg ml$^{-1}$) of LPS. Data (means ± s.d., $n = 4$) expressed as total IL-1 activity or TNF released in 24 h; n.d. = not detectable. Significance of the differences: [a]$P<0.01$ *vs* day 4, [b]$P<0.01$ *vs* controls and *vs* day 4. For full details see the Material and methods section.

animals under the influence of a variety of cancers (Kern & Norton, 1988; Theologides, 1972). Studies performed on mice bearing the MAC16 colon adenocarcinoma, a cachexia-inducing tumour, have shown an elevated nitrogen loss during early tumour growth (Beck & Tisdale, 1989). Moreover, a number of reports have pointed out that enhanced tissue protein degradation or acceleration in whole body protein turnover make a substantial contribution to the protein waste in tumour-bearing patients (Kien & Camitta, 1983, 1987; Fearon *et al.*, 1988; Melville *et al.*, 1990; O'Keefe *et al.*, 1990) and experimental animals (Tayek *et al.*, 1986, 1988; Tessitore *et al.*, 1987*a*; Beck & Tisdale, 1989; Beck *et al.*, 1991; Pain *et al.*, 1984). Consistent with these reports are our previous (Tessitore *et al.*, 1987*a*) and present observations on rats transplanted with the AH-130 Yoshida ascites hepatoma: early cachexia is associated with enhanced tissue protein catabolism. On the other hand, a comparison of fractional rates of protein synthesis and degradation in tumour cells *vs* host tissues (Tessitore *et al.*, 1987*c*) indicated that tumour cells have a definite selective advantage, particularly on the catabolic side of protein turnover. As a consequence, the

**Figure 6** Release of $PGE_2$, IL-1, and TNF from logarithmic (day 4) and stationary (day 10) AH-130 cells. Data expressed as total amounts released per $10^6$ cells/24 h. For full details see the Material and methods section. Vertical bars denote s.d.; $n = 4$. Significance of the differences: * = $P<0.01$ *vs* day 4.

**Table III** Release of IL-1 and TNF from exponential AH-130 tumour cells and peritoneal macrophages

| | *Total cells* | *Tumour cells* | *Macrophages* |
|---|---|---|---|
| IL-1 (U $10^{-6}$ cells) | 27 ± 2 | 20 ± 2 | 4.1 ± 0.06 |
| TNF (pg $10^{-6}$ cells) | 1150 ± 42 | 904 ± 23 | 325 ± 39 |

Data (means ± s.d., $n = 4$) are expressed as total IL-1 activity or TNF released in 24 h. Total cells = total ascites cells as harvested from the peritoneal cavity; tumour cells = AH-130 cells only; macrophages = peritoneal macrophages only. For full details see the Material and methods section.

**Figure 7** Release of $PGE_2$ from soleus muscle in tumour-bearing rats. Data expressed as pg of $PGE_2$ released per mg wet weight/h. For full details see the Material and methods section. Vertical bars denote s.d.; $n = 4$. Significance of the differences: * = $P <$ 0.01 *vs* day 0.

AH-130 cells clearly have the ability to grow, or at least to maintain a stationary state, though overall in the host protein catabolism predominates, particularly in some tissues. Thus, the protein metabolic perturbations in the host, while causing tissue waste, may as a consequence favour the tumour growth by providing the necessary nitrogen for protein in the tumour (cf. Beck *et al.*, 1991).

After AH-130 tumour implantation, the food intake of the host rats progressively declined. Although such a decline was sufficient to cause significant metabolic alterations in pair-fed controls, the resulting overall pattern was quite different from that in tumour hosts. Pair-fed controls developed a typical pattern of partial starvation (Waterlow *et al.*, 1978; Baccino *et al.*, 1982; Arnal *et al.*, 1987), presenting with relative loss of body weight (no body weight change compared to the gain in ad libitum-fed animals), actual protein loss from both gastrocnemius muscle and liver, and persistently reduced plasma concentrations of glucose and total free amino acids. In tumour-bearing animals, however, the body weight progressively declined, protein loss in the gastrocnemius was considerably more pronounced, total liver protein first increased at day 4 and then declined at day 10, yet remaining higher than in pair-fed controls. Moreover, tumour hosts did not develop hypoglycemia, at least until day 10, and their plasma concentration of total free amino acids, though initially decreased, returned to normal levels by day 10. Plasma lipids also showed marked differences, triglycerides and total cholesterol being increased in tumour hosts only. On the basis of such observations, hypophagia should be ruled out as a major determinant of the metabolic patterns that characterise AH-130-bearing rats, at least in early stages. Yet undernutrition undoubtedly occurs in these tumour hosts, thus adding a further component to the complexity of their metabolic perturbations. Studying a different model of cachexia, Mulligan & Tisdale (1991) have recently shown that only tumour-bearing animals that failed to adjust their food intake in the presence of metabolic disturbances underwent dramatic weight loss.

*Humoral mediators*

Altered hormonal levels as well as factors produced by host or tumour cells likely combine to cause cancer cachexia. Some of the systemic effects in cancer hosts have been ascribed to non-dialyzable circulating lipolytic and proteolytic factors produced by the tumour (Beck & Tisdale, 1987; Beck *et al.*, 1990), which is consistent with the parabiotic transfer of cachexia reported by Norton *et al.* (1985). High plasma levels of catabolic hormones, low levels of insulin, and insulin resistance of peripheral tissues are among the factors most commonly considered as a cause of cancer cachexia (Lawson *et al.*, 1982; Morrison, 1989). However, it has been suggested that hormonal changes could rather reflect an adaptation to the metabolic alterations that lead to cachexia (Svaninger *et al.*, 1987*a,b,c*). In rats bearing the AH-130 tumour a whole spectrum of hormonal alterations have been observed which may enhance tissue protein degradation: insulin was decreased (cf. Goodlad *et al.*, 1975) while counter-regulatory hormones such as corticosterone (cf. Goldberg, 1969; Odedra & Millward, 1984; Legaspi *et al.*, 1985), glucagon (cf. Kibler *et al.*, 1964) and catecholamines (cf. Van Gool *et al.*, 1984) were elevated. The high level of corticosterone at day 4 well correlated with enlarged adrenal glands and an associated hypertrophic/hyperplastic histological pattern. As an apparent exception to the hypercatabolic endocrine pattern, the low plasma concentrations of thyroid hormones might be expected to play an opposite role (Morrison *et al.*, 1988). However, a reduction of $T_3$ and $T_4$ does not necessarily imply a decrease in thyroid biological activity, as shown by Kumara-Siri *et al.* (1981): rats bearing the Walker 256 carcinoma have low concentrations of $T_3$ and $T_4$, while maintaining normal TSH concentrations.

Cytokines such as TNF and IL-1, released by macrophagic cells, have been proposed as the main mechanism for cancer cachexia (Beutler & Cerami, 1988; Mahony & Tisdale, 1988; Evans *et al.*, 1989; Fong *et al.*, 1989). Repeated administration or chronic infusion of TNF (Fong *et al.*, 1989; Michie *et al.*, 1989; Darling *et al.*, 1990) or IL-1 (Fong *et al.*, 1989) induces significant anorexia, weight loss, and loss of body protein in rats and cachexia has been observed in nude mice inoculated with CHO cells rendered TNF-producer by transfection with human TNF cDNA (Oliff *et al.*, 1987). The observations concerning the presence of TNF and IL-1 in the serum of cancer patients are inconsistent, however. Moldawer *et al.* (1988) could not detect IL-1 and TNF bioactivities in the plasma of weight-losing cancer patients; other investigators have made similar observations (Scuderi *et al.*, 1986; Waage *et al.*, 1986; Selby *et al.*, 1987; Socher *et al.*, 1988*b*). By contrast, the presence of TNF has been observed in the serum of children with acute lymphoblastic leukaemia (Saarinen *et al.*, 1990), in other cancer patients (Aderka *et al.*, 1985; Balkwill *et al.*, 1987), and in cachectic tumour-bearing rats (Stovroff *et al.*, 1989). In our studies a fairly constant elevation of TNF was detected in the plasma of AH-130-bearing rats at days 4 and 10, indicating that there was no apparent correlation with the tumour burden (at variance with previous observations on sarcoma-bearing rats [Stovroff *et al.*, 1989]).

In short term cultures the AH-130 hepatoma cells released $PGE_2$ (cf. Trevisani *et al.*, 1980; Strelkov *et al.*, 1989), IL-1-like factors, and TNF. The release of cytokines was higher in the exponential phase of growth, and thus positively correlated with the cell proliferation rate; consistently, TNF was more elevated at day 4 than at day 10 both in the ascitic fluid and in the blood plasma. Contrariwise, the release of $PGE_2$ from AH-130 cells (and its plasma concentration as well) was more elevated in the stationary phase; whether this finding implies some kind of mutual regulation between $PGE_2$ and cytokines (Goldings, 1986; Candela *et al.*, 1991) was not investigated. In addition, some IL-1 and TNF were produced by ascites macrophages, while blood monocytes from tumour-bearing rats spontaneously released TNF. The possibility that such activation of mononuclear phagocytes was triggered by cytokines (Platanias & Vogelzang, 1990; Jäättelä, 1991) originating from tumour cells should be considered. In AH-130 tumour hosts plasma $PGE_2$ was elevated and circulating TNF was detectable; although we failed to demonstrate a corresponding elevation of IL-1 activity (data not shown), the possibility that such activity was masked by inhibitors or soluble cytokine receptors in the blood plasma (Symons & Duff, 1991) cannot be excluded. This consideration also applies to TNF (Gatanaga *et al.*, 1990; Aderka *et al.*, 1991 and 1992), the levels of which might have been underestimated. Other workers (Gelin *et al.*, 1991) have made partially similar observations: TNF and IL-1 are produced by a tumour of non-lymphoid origin, the undifferentiated sarcoma MCG 101, which causes cachexia in mice and also elicits an acute phase response, yet no plasma elevation of either cytokine could be demonstrated. Our present and previous work (Tessitore *et al.*, 1987*a*), on the other hand, has clearly established that the AH-130 tumour quickly elicited in the host rat an array of changes which are among those characteristic of TNF and/or IL-1, such as increased hepatic uptake and muscular release of amino acids with reduced plasma levels of amino acids (Starnes *et al.*, 1988; Argilés *et al.*, 1989), acceleration of whole body protein turnover (Starnes *et al.*, 1988), increased degradation and depletion of muscle protein (Baracos *et al.*, 1983; Tracey *et al.*, 1988; Fong *et al.*, 1989; however, see: Kettlehut & Goldberg, 1988), transient liver hyperplasia (Feingold *et al.*, 1988; Fong *et al.*, 1989; Mealy *et al.*, 1990), augmented plasma levels of triglycerides (Feingold & Grunfeld, 1987; Starnes *et al.*, 1988; Grunfeld *et al.*, 1989), increased hepatic synthesis and serum levels of cholesterol (Feingold & Grunfeld, 1987). TNF or IL-1 can also activate a variety of other mechanisms and cause the elevation of plasma mediators such as cathecholamines and glucagon (Starnes *et al.*, 1988; Rivier *et al.*, 1989), cortisol or corticosterone and ACTH (Del Rey & Besedowsky, 1987; Starnes *et al.*, 1988; Kehrer *et al.*, 1988; Tracey *et al.*, 1988; Argilés *et al.*, 1989; Rivier *et al.*, 1989; Sharp *et al.*, 1989) and $PGE_2$ (Dayer *et al.*, 1985). By contrast, serum $T_3$ and $T_4$ decline after TNF (Imamura *et al.*, 1988) and were decreased in AH-130 tumour-bearers.

The role of cytokines in cancer cachexia may appear questionable in view of the observation that the administration of a single cytokine renders most animals refractory to its effects (Mahony & Tisdale, 1988; Socher *et al.*, 1988*a*; Stovroff *et al.*, 1989; Tracey *et al.*, 1988; Grunfeld *et al.*, 1989; Darling *et al.*, 1990). This would imply, at least, that single cytokines must act in concert with other cytokines or factors (Michie *et al.*, 1989) for cancer cachexia to develop. On the other hand, cachexia could be attenuated in MCG 101 sarcoma-bearing mice with anti-TNF antibodies (Sherry *et al.*, 1989) or in rats transplanted with a methylcholanthrene-induced sarcoma by eliciting tolerance to TNF through repeated administration of this cytokine at low dosage (Sheppard *et al.*, 1990). Besides the MCG 101 sarcoma (Gelin *et al.*, 1991) and the AH-130 hepatoma, also the Ehrlich ascites tumour cells produce TNF and circulating TNF can be detected in the host mice (Tessitore L., Costelli, P., Baccino, F.M., unpublished data). These observations are of special interest since cytokine production by tumours was previously known only for leukaemic cells (e.g., Platanias & Vogelzang, 1990; Aguilar-Santelises *et al.*, 1991). Thus for a few experimental tumours, among those having the ability to elicit a rapidly progressive cachexia, the host-wasting properties seem associated, at least in part, with their ability to release significant amounts of cytokines, such as IL-1 and TNF (and possibly of other factors such as $PGE_2$).

### *Concluding remarks*

The primary causative factors involved in the profound metabolic perturbations that lead to cancer-induced cachexia remain largely elusive as yet. Many different mechanisms have been cited in different situations, either in humans or in experimental models. In the present study a number of potentially relevant parameters have been found to be altered in rats bearing the AH-130 Yoshida ascites hepatoma and further work is needed to clarify which, among them, may have an initiating role. In any event, there seems no doubt that cancer cachexia in the present model reflects complex homeostatic perturbations and develops through the interplay of multiple factors (hormone changes, production of cytokines, hypophagia), virtually all which seem to converge in forcing protein metabolism, particularly in skeletal muscle, into a hypercatabolic state.

Work supported by the Ministero dell'Università e della Ricerca Scientifica e Tecnologica, Roma, the Consiglio Nazionale delle Ricerche (Special Project A.C.R.O.), and the Associazione Italiana per la Ricerca sul Cancro, Milano. Presented in part at the Italy-USA Binational Symposium on 'Protein Metabolism in Aging' and 'Intervention in Aging', San Miniato and Pisa, April 1989 (Baccino *et al.*, 1989). The authors thank Dr J.S. Amenta (Pittsburgh, PA, USA) for useful discussion and critical review of the manuscript.

### References

ADERKA, D., FISHER, S., LEVO, Y., HOLTMANN, H., HAHN, T. & WALLACH, D. (1985). Cachectin/tumour-necrosis-factor production by cancer patients. *Lancet*, **ii**, 1190.

ADERKA, D., ENGELMANN, H., HORNIK, V., SKORNICK, Y., LEVO, Y., WALLACH, D. & KUSHTAI, G. (1991). Increased serum levels of soluble receptors for tumor necrosis factor in cancer patients. *Cancer Res.*, **51**, 5602–5607.

ADERKA, D., ENGELMANN, H., MAOR, Y., BRAKEBUSCH, C. & WALLACH, D. (1992). Stabilization of the bioactivity of tumor necrosis factor by its soluble receptors. *J. Exp. Med.*, **175**, 323–329.

AGUILAR-SANTELISES, M., MAGNUSSON, R., SVENSON, S.B., LOFTENIUS, A., ANDERSSON, B., MELLSTEDT, H. & JONDAL, M. (1991). Expression of interleukin-1α, interleukin-1β and interleukin 6 in chronic B lymphocytic leukaemia (B-CLL) cells from patients at different stages of disease progression. *Clin. Exp. Immunol.*, **84**, 422–428.

ANGELI, A., BISBOCCI, D., MELÔ, F,. FRAIRIA, R. & GAIDANO, G. (1975). Relative competition of corticosterone, cortisol, cortisone, 11-deoxycortisol and prednisolone with [1,2-$^3$H]-cortisol in various protein-binding radioassay systems. *Clin. Chim. Acta (Amst.)*, **61**, 279–286.

ARGILÉS, J.M., LOPEZ-SORIANO, J., WIGGINS, D. & WILLIAMSON, D.H. (1989). Comparative effects of tumour necrosis factor-α (cachectin), interleukin-1-β and tumour growth on amino acid metabolism in the rat *in vivo*. *Biochem. J.*, **261**, 357–362.

ARNAL, M., OBLED, C., ATTAIX, D., PATUREAU-MIRAND, P. & BONIN, D. (1987). Dietary control of protein turnover. *Diab. Met.*, **13**, 630–642.

BACCINO, F.M., TESSITORE, L., CECCHINI, G., MESSINA, M., ZURETTI, M.F., BONELLI, G., GABRIEL, L. & AMENTA, J.S. (1982). Control of cell protein catabolism in rat liver: effect of starvation and cycloheximide. *Biochem. J.*, **206**, 395–405.

BACCINO, F.M., TESSITORE, L., BONELLI, G., AUTELLI, R., COSTELLI, P., ISIDORO, C. & AMENTA, J.S. (1989). Protein turnover regulations and mechanisms in neoplastic cells and host tissues. Italy - USA. *Binational Symposium on 'Protein metabolism in Aging' and 'Intervention in Aging'*, San Miniato and Pisa, 2–6 April 1989, Abstracts p. 15.

BALKWILL, F., BURKE, F., TALBOT, D., TAVERNIER, J., OSBORNE, R., NAYLOR, S., DURBIN, H. & FIERS, W. (1987). Evidence for tumour necrosis factor/cachectin production in cancer. *Lancet*, **28**, 1229–1231.

BARACOS, V., RODEMANN, P., DINARELLO, C.A. & GOLDBERG, A.L. (1983). Stimulation of muscle protein degradation and prostaglandin $E_2$ release by leukocytic pyrogen (interleukin-1). *New Eng. J. Med.*, **308**, 553–558.

BECK, S.A. & TISDALE, M.J. (1987). Production of lipolytic and proteolytic factors by a murine tumor-producing cachexia in the host. *Cancer Res.*, **47**, 5919–5923.

BECK, S.A. & TISDALE, M.J. (1989). Nitrogen excretion in cancer cachexia and its modification by a high fat diet in mice. *Cancer Res.*, **49**, 3800–3804.

BECK, S.A., SMITH, K.L. & TISDALE, M.J. (1991). Anticachectic and antitumour effect of eicosapentaenoic acid and its effects on protein turnover. *Cancer Res.*, **51**, 6089–6093.

BEUTLER, B. & CERAMI, A. (1988). The history, properties, and biological effects of cachectin. *Biochemistry*, **27**, 7575–7582.

BOZZETTI, F., AMMATUNA, M., MIGLIAVACCA, S., BONALUMI, M.G., FACCHETTI, G., PUPA, A. & TERNO, G. (1987). Total parenteral nutrition prevents further nutritional deterioration in patients with cancer cachexia. *Ann. Surg.*, **205**, 138–143.

CANDELA, M., BARKER, S.C. & BALLOU, L.R. (1991). Sphingosine synergistically stimulates tumor necrosis factor α-induced prostaglandin $E_2$ production in human fibroblasts. *J. Exp. Med.*, **174**, 1363–1369.

CANNON, J.G. & DINARELLO, C.A. (1985). Increased plasma interleukin-1 activity in women after ovulation. *Science*, **227**, 1247–1249.

DARLING, G., FRAKER, D.L., JENSEN, C.J., GORSCHBOTH, C.M. & NORTON, J.A. (1990). Cachectic effects of recombinant human tumor necrosis factor in rats. *Cancer Res.*, **50**, 4008–4013.

DAYER, J.M, BEUTLER, B. & CERAMI, A. (1985). Cachectin/tumor necrosis factor stimulates collagenase and prostaglandin $E_2$ production by human synovial cells and dermal fibroblasts. *J. Exp. Med.*, **162**, 2163–2168.

DEL REY, A. & BESEDOWSKY, H. (1987). Interleukin-1 affects glucose homeostatis. *Am. J. Physiol.*, **253**, R794–R798.

DESSÌ, S., BATETTA, B., ANCHISI, C., PANI, P., COSTELLI, P., TESSITORE, L. & BACCINO, F.M. (1992). Cholesterol metabolism during the growth of a rat ascites hepatoma. *Br. J. Cancer*, **66**, 787–793.

EVANS, R.D., ARGILES, J.M. & WILLIAMSON, D.H. (1989). Metabolic effects of tumour necrosis factor-α (cachectin) and interleukin-1. *Clin. Sci.*, **77**, 357–364.

FEARON, K.C.H., HANSELL, D.T., PRESTON, T., PLUMB, J.A., DAVIES, J., SHAPIRO, D., SHENKIN, A., CALMAN, K.C. & BURNS, H.J.G. (1988). Influence of whole body protein turnover rate on resting energy expenditure in patients with cancer. *Cancer Res.*, **48**, 2590–2595.

FEINGOLD, K.R. & GRUNFELD, C. (1987). Tumor necrosis factor-alpha stimulates hepatic lipogenesis in the rat *in vivo*. *J. Clin. Invest.*, **80**, 184–190.

FLICK, D.A. & GIFFORD, G.E. (1984). Comparison of *in vitro* cell cytotoxic assays for tumor necrosis factor. *J. Immunol. Methods*, **68**, 167–175.

FONG, Y., MOLDAWER, L.L., MARANO, M., WEI, H., BARBER, A., MANOGUE, K., TRACEY, K.J., KUO, G., FISHMAN, D.A., CERAMI, A. & LOWRY, S.F. (1989). Cachectin/TNF or IL-1α induces cachexia with redistribution of body proteins. *Am. J. Physiol.*, **256**, R659–R665.

GATANAGA, T., HWANG, C., KOHR, W., CAPPUCCINI, F., LUCCI, J.A., JEFFES, E.W., LENTZ, R., TOMICH, J., YAMAMOTO, R.S. & GRANGER, G. (1990). Purification and characterization of an inhibitor (soluble tumor necrosis factor receptor) for tumor necrosis factor and lymphotoxin obtained from the serum ultrafiltrates of human cancer patients. *Proc. Natl Acad. Sci. USA*, **87**, 8781–8784.

GELIN, J., MOLDAWER, L.L., LÖNNROTH, C., SHERRY, B., CHIZZONITE, R. & LUNDHOLM, K. (1991). Role of endogenous tumor necrosis factor α and interleukin 1 for experimental tumor growth and the development of cancer cachexia. *Cancer Res.*, **51**, 415–421.

GOLDBERG, A.L. (1969). Protein turnover in skeletal muscle. II. Effect of denervation and cortisone on protein catabolism in skeletal muscle. *J. Biol. Chem.*, **244**, 3223–3229.

GOLDINGS, E.A. (1986). Regulation of B cell tolerance by macrophage-derived mediators: antagonistic effects of prostaglandin $E_2$ and interleukin 1. *J. Immunol.*, **136**, 817–822.

GOODLAD, G.A.J., MITCHELL, A.J.H., MCPHAIL, L. & CLARK, C.M. (1975). Serum insulin and somatomedin levels in the tumour-bearing rat. *Eur. J. Cancer*, **11**, 733–737.

GRUNFELD, C., WILKING, H., NEESE, R., GAVIN, R.A., MOSER, A.H., GULLI, R., KERALA SERIO, M. & FEINGOLD, K.R. (1989). Persistence of hypertriglyceridemic effect of tumor necrosis factor despite development of tachyphylaxis to its anorectic/cachectic effects in rats. *Cancer Res.*, **49**, 2554–2560.

IMAMURA, H., SATO, K., OSAWA, K., SHIZUME, K. & KAWAKAMI, M. (1988). Tumor necrosis factor-alpha (cachectin) induces low $T_3$ and low $T_4$ serum concentrations in mice. *8th International Congress of Endocrinology*, p. 325. Kyto, July 17–23.

JÄÄTTELÄ, M. (1991). Biologic activities and mechanisms of action of tumour necrosis factor-α/cachectin. *Lab. Invest.*, **64**, 724–742.

KERN, K.A., NORTON, J.A. (1988). Cancer cachexia. *J. Parent. Ent Nutr.*, **12**, 286–298.

KEHRER, P., TURNHILL, D., DAYER, J.M., MULLER, A.F. & GAILLARD, R.C. (1988). Human recombinant interleukin-1beta and -alpha, but not recombinant tumor necrosis fractor alpha stimulate ACTH release from rat anterior pituitary cells *in vitro* in a prostaglandin $E_2$ and cAMP independent manner. *Neuroendocrinology*, **48**, 160–166.

KETTLEHUT, I.C. & GOLDBERG, A.L. (1988). Tumor necrosis factor can induce fever in rats without activating protein breakdown in muscle or lipolysis in adipose tissue. *J. Clin. Invest.*, **81**, 1384–1389.

KIBLER, R.F., TAYLOR, W.R. & MEYER, J.D. (1964). The effect of glucagon on net splanchnic balances of glucose, amino acids, nitrogen, urea, ketones, and oxygen in man. *J. Clin. Invest.*, **43**, 904–915.

KIEN, C.L. & CAMITTA, B.M. (1983). Increased whole-body protein turnover in sick children with newly diagnosed leukemia or lymphoma. *Cancer Res.*, **43**, 5586–5592.

KIEN, C.L. & CAMITTA, B.M. (1987). Close association of accelerated rates of whole body protein turnover (synthesis and breakdown) and energy expenditure in children with newly diagnosed acute lymphoyctic leukaemia. *J. Parent. Ent. Nutr.*, **11**, 129–134.

KUMARA-SIRI, M.H., LEE, K. & SURKS, M.I. (1981). Regulation of thyrotropin secretion in rats bearing the Walker 256 carcinoma. *Endocrinol.*, **109**, 1760–1768.

LAGOPOULOS, L., SUNAHARA, G., WÜRZNER, H.P., FLIESEN, T. & STALDER, R. (1991). The correlation of body growth with diethylnitrosamine-induced hepatocarcinogenesis in relation to serum insulin and somatomedin-C. *Carcinogenesis*, **12**, 211–215.

LAWSON, D.H., RICHMOND, A., NIXON, D.W. & RUDMAN, D. (1982). Metabolic approaches to cancer cachexia. *Annu. Rev. Nutr.*, **2**, 277–301.

LEGASPI, A., ALBERT, J.D. & CALVANO, S.E. (1985). Proteolysis of skeletal muscle in response to acute elevation of plasma cortisol in man. *Surg. Forum*, **36**, 16–18.

MAHONY, S.M. & TISDALE, M.J. (1988). Induction of weight loss and metabolic alterations by human recombinant tumour necrosis factor. *Br. J. Cancer*, **58**, 345–349.

MEALY, K., VAN LANSCHOT, J.J., ROBINSON, B.G., ROUNDS, J. & WILMORE, D.W. (1990). Are the catabolic effects of tumor necrosis factor mediated by glucocorticoids? *Arch. Surg.*, **125**, 42–48.

MELVILLE, S., MCNURLAN, M.A., GRAHAM CALDER, A. & GARLICK, P.J. (1990). Increased protein turnover despite normal energy metabolism and responses to feeding in patients with lung cancer. *Cancer Res.*, **50**, 1125–1131.

MICHIE, H.R., SHERMAN, M.L., SPRIGGS, D.R., ROUNDS, J., CHRISTIE, M. & WILMORE, D.W. (1989). Chronic TNF infusion causes anorexia but not accelerated nitrogen loss. *Ann. Surg.*, **209**, 19–24.

MOLDAWER, L.L., DROTT, C. & LUNDHOLM, K. (1988). Monocytic production and plasma bioactivities of interleukin-1 and tumour necrosis factor in human cancer. *Eur. J. Clin. Invest.*, **18**, 486–492.

MOORE, A. & STEIN, R.A. (1951). Chromatography of amino acids on sulfonated polystyrene resins. *J. Biol. Chem.*, **192**, 663–666.

MORRISON, S.D., MOLAY, J.F. & NORTON, J.A.(1984). Contribution of inert mass to experimental cancer cachexia in rats. *J. Natl Cancer Inst.*, **73**, 991–998.

MORRISON, W.L., GIBSON, J.N.A., JUNG, R.T. & RENNIE, M.J. (1988). Skeletal muscle and whole body protein turnover in thyroid disease. *Eur. J. Clin. Invest.*, **18**, 62–68.

MORRISON, S.D. (1989). Cancer cachexia. In *Influence of Tumor Development on the Host*, Liotta, A.L. (ed), pp. 176–213. Kluwer Acadmic Publisher: Netherlands.

MULLIGAN, H.D. & TISDALE, M.J. (1991). Lipogenesis in tumour and host tissues in mice bearing colonic adenocarcinomas. *Br. J. Cancer*, **63**, 719–722.

NIGAN, S., BECKER, R., ROSENDHAL, V., HAMMERSTEIN, J., BENEDETTO, C., BARBERO, M. & SLATER, T.F. (1985). *Prostaglandins*, **29**, 513–515.

NORTON, J.A., MOLEY, J.F., GREEN, M.V., CARSON, R.E. & MORRISON, S.D. (1985). Parabiotic transfer of cancer anorexia/cachexia in male rats. *Cancer Res.*, **48**, 654–657.

ODEDRA, B.R. & MILLWARD, D.J. (1984). Effect of corticosterone treatment on muscle protein turnover in adrenalectomized rats and diabetic rats maintained on insulin. *Biochem. J.*, **204**, 663-672.

O'KEEFE, S.J.D., ODGEN, J,. RAMJEE, G. & RUND, J. (1990). Contribution of elevated protein turnover and anorexia to cachexia in patients with hepatocellular carcinoma. *Cancer Res.*, **50**, 1226-1230.

OLIFF, A., DEFEO-JONES, D., BOYER, M., MARTINEZ, D., KEIFER, D., VUOCOLO, G., WOLFE, A. & SOCHER, S.H. (1987). Tumors secreting human TNF/cachectin induce cachexia in mice. *Cell*, **50**, 555-563.

PAIN, V.M., RANDALL, D.P. & GARLICK, P.J. (1984). Protein synthesis in liver and skeletal muscle of mice bearing an ascites tumor. *Cancer Res.*, **44**, 1054-1057.

PLATANIAS, L.C. & VOGELZANG, N.J. (1990). Interleukin-1: biology, pathophysiology, and clinical prospects. *Am. J. Med.*, **89**, 621-629.

PISTERS, P.W.T. & BRENNAN, M.F. (1990). Amino acid metabolism in human cancer cachexia. *Annu. Rev. Nutr.*, **10**, 107-132.

RIVIER, C., VALE, W. & BROWN, M. (1989). In the rat, interleukin-1α and -β stimulate adrenocorticotropin and catecholamine release. *Endocrinology*, **125**, 3096-3102.

RODEMANN, H.P. & GOLDBERG, A.L. (1982*a*). Arachidonic acid, prostaglandin $E_2$ and $F_{2\alpha}$ influence rates of protein turnover in skeletal and cardiac muscle. *J. Biol. Chem.*, **257**, 1632-1638.

RODEMANN, H.P., WAXMAN, L. & GOLDBERG, A.L. (1982*b*). The stimulation of protein degradation by $Ca^{2+}$ is mediated by $PGE_2$ and does not require the calcium-activated protease. *J. Biol. Chem.*, **257**, 8716-8723.

SAARINEN, U.M., KOSKELO, E.K., TEPPO, A.M. & SIIMES, M.A. (1990). Tumor necrosis factor in children with malignancies. *Cancer Res.*, **50**, 592-595.

SABATINI, M., BRENDAN, B., AUFDEMORTE, T., BONEWALD, L. & MUNDY, G.R. (1988). Infusions of recombinant human interleukins 1α and 1β cause hypercalcemia in normal mice. *Proc. Natl Acad. Sci. USA*, **85**, 5235-5239.

SABATINI, M., YATES, A.J., GARRETT, I.R. & 4 others (1990). Increased production of tumor necrosis factor by normal immune cells in a model of the humoral hypercalcemia of malignancy. *Lab. Invest.*, **63**, 676-682.

SCUDERI, P., STERLING, K.E., LAM, K.S., FINLEY, P.R., RYAN, K.J., RAY, C.G., PETERSEN, E., SLYMEN, D.J. & SALMON, S.E. (1986). Raised serum levels of tumor necrosis factor in parasitic infections. *Lancet*, **ii**, 1364-1365.

SELBY, P., HOBBS, S., VINER, C., JACKSON, E., JONES, A., NEWELL, D., CALVERT, A.H., MCELVAIN, T., FEARON, K., HUMPREYS, J. & SHIGA, T. (1987). Tumour necrosis factor in man: clinical and biological observations. *Br. J. Cancer*, **56**, 803-808.

SHAPOT, V.S. (1979). On the multiform relationships between the tumor and the host. *Adv. Cancer Res.*, **30**, 89-143.

SHARP, B.M., SHANNON, M.G., PETERSON, P.K., NEWTON, R., CHAO, C. & MCALLEN, K. (1989). Tumor necrosis factor-α is a potent ACTH secretagogue: comparison to interleukin-1β. *Endocrinology*, **124**, 3131-3137.

SHEPPARD, B.C., VENZON, D., FRAKER, D.L., LANGSTEIN, H.N., JENSEN, C.J. & NORTON, J.A. (1990). Prolonged survival of tumor-bearing rats with respetitive low-dose recombinant tumor necrosis factor. *Cancer Res.*, **50**, 3928-3933.

SHERMAN, M.L., SPRIGGS, D.R., ARTHUR, K.A., IMAMURA, K., FREI, E. & KUFE, D.W. (1988). Recombinant human tumor necrosis factor administered as a five-days continuous infusion in cancer patients: phase I toxicity and effects on lipid metabolism. *J. Clin. Oncol.*, **6**, 344-350.

SHERRY, B.A., GELIN, J., FONG, Y., MARANO, M., WEI, H., CERAMI, A., LOWRY, S.F., LUNDHOLM, K. & MOLDAWER, L.L. (1989). Anticachectin/tumor necrosis factor-α antibodies attenuate development of cachexia in tumor model. *FASEB J.*, **3**, 1956-1962.

SOCHER, S.H., FREIDMAN, A. & MARTINEZ, D. (1988*a*). Recombinant tumor necrosis factor induces acute reductions in food intake and body weight in mice. *J. Exp. Med.*, **167**, 1957-1962.

SOCHER, S.H., MARTINEZ, D., CRAIG, J.B., KUHN, J.G. & OLIFF, A. (1988*b*). Tumour necrosis factor not detectable in patients with cancer cachexia. *J. Natl. Cancer Inst.*, **80**, 595-598.

STARNES, H.F., WARREN, R.S., JEEVANANDAM, M., GABRILOVE, J.L., LARCHIAN, W., OETTGEN, H.F. & BRENNAN, M.F. (1988). Tumor necrosis factor and the acute metabolic response to tissue injury in man. *J. Clin. Invest.*, **82**, 1321-1325.

STOVROFF, M.C., FRAKER, D.L. & NORTON, J.A. (1989). Cachectin activity in the serum of cachectic, tumor-bearing rats. *Arch. Surg.*, **124**, 94-99.

STRELKOV, A.B., FIELDS, A.L.A. & BARACOS, V.E. (1989). Effects of systemic inhibition of prostaglandin production on protein metabolism in tumor-bearing rats. *Am. J. Physiol.*, **257**, C261-C269.

SVANINGER, G., DROTT, C. & LUNDHOLM, K. (1987*a*). Role of insulin in the development of cancer cachexia in nongrowing sarcoma-bearing mice: special reference to muscle wasting. *J. Natl Cancer Inst.*, **78**, 943-950.

SVANINGER, G., GELIN, J. & LUNDHOLM, K. (1987*b*). Tumor-host wasting not explained by adrenal hyperfunction in tumor-bearing animals. *J. Natl Cancer Inst.*, **79**, 1135-1141.

SVANINGER, G., ISAKSSON, O. & LUNDHOLM, K. (1987*c*). Growth hormone and experimental cancer cachexia. *J. Natl Cancer Inst.*, **79**, 1359-1365.

SYMONS, J.A. & DUFF, G.W. (1991). Purification and characterisation of a natural soluble receptor for interleukin-1. *Cytokine*, **3**, 478.

TANAKA, Y., TANAKA, T. & ISHITSUKA, H. (1989). Antitumor activity of indomethacin in mice bearing advanced colon 26 carcinoma compared with those with early transplants. *Cancer Res.*, **49**, 5935-5939.

TASHJIAN, A.H., VEOLKEL, E.F., MARIBETH, L., GOAD, D., BOSMA, T. & LEVINE, L. (1974). Tumor necrosis factor-α (cachectin) stimulates bone resorption in mouse calvaria via a prostaglandin-mediated mechanism. *Endocrinol.*, **120**, 2029-2033.

TAYEK, J.A., ISTFAN, N.W., JONES, C.T., HAMAWY, K.J., BISTRIAN, B.R. & BLACKBURN, G.L. (1986). Influence of the Walker 256 carcinosarcoma on muscle, tumor and whole-body protein synthesis and growth rate in the cancer-bearing rat. *Cancer Res.*, **46**, 5649-5654.

TAYEK, J.A., BLACKBURN, G.L. & BISTRIAN, B.R. (1988). Alterations in whole body, muscle, liver, and tumor tissue protein synthesis and degradation in Novikoff hepatoma and Yoshida sarcoma tumor growth studied *in vivo*. *Cancer Res.*, **48**, 1554-1558.

TESSITORE, L., BONELLI, G. & BACCINO, F.M. (1987*a*). Early development of protein metabolic perturbations in the liver and skeletal muscle of tumour-bearing rats. A model system for cancer cachexia. *Biochem. J.*, **241**, 153-158.

TESSITORE, L., MATERA, L., BONELLI, G., BACCINO, F.M. & DIANZANI, M.U. (1987*b*). Aliphatic aldheydes inhibit the proliferative response of human peripheral blood lymphocytes to phytohemagglutinin and alloantigens. *Chem. Biol. Interact.*, **62**, 217-226.

TESSITORE, L., BONELLI, G., CECCHINI, G., AMENTA, J.S. & BACCINO, F.M. (1987*c*). Regulation of protein turnover versus growth state: ascites hepatoma as a model for studies both in the animal and *in vitro*. *Arch. Biochem. Biophys.*, **255**, 372-384.

THEOLOGIDES, A. (1972). Pathogenesis of cachexia in cancer, a review and a hypothesis. *Cancer*, **20**, 484-488.

TRACEY, K.J., WEI, H., MANOGUE, K.R., FONG, Y., HESSE, D.G., NGUYEN, H.T., COTRAN, R.S., CERAMI, A. & LOWRY, S.F. (1988). Cachectin/TNF induces cachexia, anemia, and inflammation. *J. Exp. Med.*, **167**, 1211-1227.

TREVISANI, F., FERRETTI, E., CAPUZZO, A. & TOMASI, V. (1980). Elevated levels of prostaglandin $E_2$ in Yoshida hepatoma and the inhibition of tumour growth by non-steroidal anti-inflammatory drugs. *Br. J. Cancer*, **41**, 341-347.

VAN GOOL, J., BOERS, W. & SALA, M. (1984). Glucocorticoids and catecholamines as mediators of acute-phase proteins, especially rat α-macrofoetoprotein. *Biochem. J.*, **220**, 125-132.

WAAGE, A., ESPEVIK, T. & LAMVIK, J. (1986). Detection of tumour necrosis factor-like cytotoxicity in serum from patients with septicaemia but not from untreated cancer patients. *Scand. J. Immunol.*, **24**, 739-743.

WATERLOW, J.C., GARLICK, P.J. & MILLWARD, D.J. (1978). *Protein turnover in mammalian tissues and in the whole body*. North-Holland, Amsterdam.

WATERLOW, J.C. (1984). Protein turnover with special reference to man. *Quart. J. Exp. Physiol.*, **69**, 409-438.